# Antibody-Based Assays for Phenotyping of Extracellular Vesicles

**DOI:** 10.1155/2015/524817

**Published:** 2015-12-03

**Authors:** Lotte Hatting Pugholm, Anne Louise Schacht Revenfeld, Evo Kristina Lindersson Søndergaard, Malene Møller Jørgensen

**Affiliations:** Department of Clinical Immunology, Aalborg University Hospital, 9000 Aalborg, Denmark

## Abstract

Extracellular vesicles (EVs) are a heterogeneous population of membrane-enclosed vesicles. EVs are recognized as important players in cell-to-cell communication and are described to be involved in numerous biological and pathological processes. The fact that EVs are involved in the development and progression of several diseases has formed the basis for the use of EV analysis in a clinical setting. As the interest in EVs has increased immensely, multiple techniques have been developed aiming at characterizing these vesicles. These techniques characterize different features of EVs, like the size distribution, enumeration, protein composition, and the intravesicular cargo (e.g., RNA). This review focuses on techniques that exploit the specificity and sensitivity associated with antibody-based assays to characterize the protein phenotype of EVs. The protein phenotype of EVs can provide information on the functionality of the vesicles and may be used for identification of disease-related biomarkers. Thus, protein profiling of EVs holds great diagnostic and prognostic potential.

## 1. Introduction

In 1967 Wolf described extracellular vesicles (EVs) as an unwanted contamination of a platelet preparation [1]. For long EVs were considered artifacts or fragments of degenerated or dead cells; however, as a consequence of an immensely increased interest in these vesicles, during the past decades, it is now recognized that EVs are involved in numerous physiological processes, but also in pathophysiological processes [2–4]. EVs are considered as a pivotal part of the intercellular environment and may act as important players in cell-to-cell communication. The fact that EVs are involved in the development and progression of several diseases has formed the basis for the use of EV analysis in a clinical setting and envisions a great potential for using EVs as disease-related biomarkers. In recent years, several techniques have been developed with the aim of identifying the molecular composition, the cellular origin, and the vesicular cargo of EVs. Such techniques provide the opportunity to employ EV analysis as a part of a diagnostic and a prognostic platform. A selection of the antibody-based techniques will be reviewed here.

### 1.1. Classification of EVs

**Figure 1:**
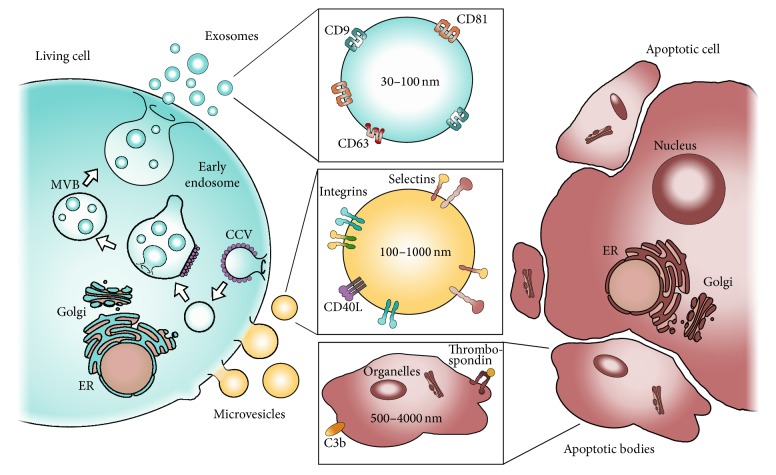
An overview of the three major EV subtypes. The biogenesis of these different subtypes of EVs is illustrated. Exosomes (blue) and MVs (yellow) are released from a living cell (blue) by fusion of multivesicular bodies (MVBs) with the plasma membrane or budding of the plasma membrane, respectively. Apoptotic bodies are released from a cell undergoing apoptosis (red). Selected physical and phenotypical differences between the three subtypes are depicted.

EVs are a heterogeneous population of membrane-enclosed vesicles that can be divided into a number of subpopulations based on specific characteristics like their size, biogenesis, cellular origin, protein composition, and biological function. So far, the scientific community has not reached consensus regarding the nomenclature of EVs, but using the biogenesis as a classification tool, EVs can be divided into three major subtypes, namely, exosomes, microvesicles (MVs), and apoptotic bodies (Figure 1) [5, 6]. As many of the properties of EVs have been reviewed in detail elsewhere, the following section states the overall characteristics of these three EV subtypes.

#### 1.1.1. Exosomes

Exosomes are the subtype of EVs that have received most attention during the past years. Exosomes are of endocytic origin and formed by invagination of the endosomal membrane, which forms vesicles inside the endosomal compartment, generating multivesicular bodies (MVBs). When the MVBs fuse with the plasma membrane exosomes are released into the extracellular space (blue, Figure 1) [7]. The release of exosomes depends on cytoskeleton activation but not on Ca^2+^ influx into the cell [8]. Due to the biogenesis of the exosomes, the orientation of the exosomal membrane proteins is similar to that of the plasma membrane. In addition to a similar orientation, the lipid composition of the exosomal membrane is similar to that of the plasma membrane and contains cholesterol, ceramide, and phosphatidylserine (PS) along with several proteins that currently are used to identify exosomes [9, 10]. These include proteins involved in the MVB formation machinery (e.g., Alix and TSG101), proteins from the membrane and fusion machinery (e.g., GTPases, annexins, and flotillins), and the tetraspanins (CD9, CD63, and CD81) (Figure 1) [7, 10, 11]. Furthermore, it has been described that exosomes display saccharide groups on their surface [12]. The different markers are not ubiquitously present on all exosomes but are present on a large proportion of these vesicles, which is why they are generally accepted as exosomal markers. In addition to the exosomal proteins, exosomes often present a molecular composition that reflects the molecular signature of the parent cells. Importantly, accumulating data indicate that the molecular contents of exosomes do not result from casual sampling of molecules from the parental cell but arise from an ability to load specific molecules into exosomes [13]. It is also reported that exosomes can contain significant amounts of RNA, including miRNAs, noncoding RNAs, and mRNAs [14–17]. The group of exosomes comprises small membrane vesicles varying from 30 to 100 nm in diameter and with a density range from 1.13 to 1.19 g/mL [7, 10, 18].

#### 1.1.2. Microvesicles

Microvesicles are formed from outward budding of the plasma membrane, releasing the MVs directly into the extracellular space (yellow, Figure 1). This process depends on cytoskeleton activation, as well as an increase in intracellular Ca^2+^ [8, 19–21]. Most MVs present PS in the outer leaflet of the membrane and even though this feature has often been used to isolate and identify MVs in biological samples, several studies indicate that PS may only be present on a subset of MVs [10, 20, 22–27]. Furthermore, studies have identified markers like CD40 ligand, adenosine diphosphate ribosylation factor 6, and several integrins and selectins on MVs [10, 19, 21, 28]. The intravesicular cargo of MVs includes membrane and cytosolic proteins, mRNAs, and miRNAs [19, 28]. MVs are a heterogeneous population of vesicles and are quite large (100 to 1000 nm) compared to exosomes [10, 29]. As a group, the density of MVs is currently undescribed; however, Ettelaie et al. have reported that MVs positive for tissue-factor (TF) have a relatively low density (1.03–1.08 g/mL) [30].

#### 1.1.3. Apoptotic Bodies

Apoptotic bodies are released when cells become apoptotic and they are formed by blebbing of the plasma membrane, releasing the apoptotic bodies straight into the extracellular space (red, Figure 1) [20]. Similar to the other subtypes of EVs, apoptotic bodies present PS in the outer leaflet of the lipid bilayer. In addition, they present thrombospondin and complement component C3b, which can be used for identifying this specific subtype [31]. Furthermore, apoptotic bodies can be distinguished from the other EV subtypes by containing organelles, DNA fragments, and histones as part of the vesicular cargo in addition to proteins and other molecules from the cytosol of the parent cell. This complex intravesicular structure is supported by transmission electron microscopy (TEM) in which the morphology of apoptotic bodies is more heterogeneous than the other EVs populations [19, 20, 32, 33]. Apoptotic bodies are the largest vesicle type of the three EV subtypes described here and range in size from approximately 500 to 4000 nm [32, 34]. The density of apoptotic vesicles is 1.16 to 1.28 g/mL, which is partly overlapping with exosomes [18].

### 1.2. Clinical Aspects of EVs

EVs are recognized as important players in cell-to-cell communication by binding and fusing with recipient cells upon having travelled either short or long distance in the body [35–38]. In fact, EVs and especially exosomes are considered as specifically secreted vesicles targeting selected recipient cells. Nonetheless, the exact mechanisms by which EVs act on one cell over another currently remain undefined [11, 39]. EVs are present in varying numbers in different biological fluids, for example, blood, urine, cerebrospinal fluid, and saliva, which enable easy and noninvasive (urine, saliva, and breast milk) or minimally invasive (blood) access to EVs [40–44]. Studies have shown that the quantity and the molecular composition of EVs shed from various cell types differ considerably. However, cells continuously release EVs into the extracellular space and several studies have shown that this release increases upon cellular activation and during pathophysiological conditions [2–4, 45]. Hence, enumeration of EVs in body fluids may provide indications of an ongoing pathological process. Furthermore, the specific molecular composition of EVs facilitates the ability to use EVs for detection of disease-related biomarkers. Identification of such markers by using EV analysis offers the ability to distinguish sick from healthy individuals and contains immense potential for early diagnosis of diseases. Recent technological developments allow for isolation, capturing, and characterization of EVs. Besides the specific membrane protein composition, EVs are enriched for nucleic acids, in particular small RNAs, like miRNAs and mRNAs, but also RNAs that generally exist in complex with proteins [14, 17, 19]. The intravesicular cargo is protected by degradation, which enables transport (and transfer) of otherwise degradable molecules over long distances. Delivery of functional cargo may induce alterations in gene expression, leading to functional changes in the recipient cell [16, 39, 46]. This inherent ability of EVs to carry cargo can be exploited in clinical settings, including drug delivery.

The apparent role of EVs in pathological processes forms the basis of extending EV analysis beyond basic research and into a clinical and therapeutic setting. The application areas include identification of disease-related biomarkers for diagnostic and prognostic purposes. In addition, EVs may be exploited in a therapeutic context, like regenerative medicine, cancer vaccines, and drug delivery.

#### 1.2.1. Diagnostic and Prognostic Potential of EVs

The application of EVs in a diagnostic and prognostic setting is based on the presence of disease-related proteins or RNAs on the surface or as the cargo of EVs. As EVs contain proteins and RNAs from the parent cell, the molecular composition of EVs will most likely reflect an aberrant parent cell. The potential of EVs as clinical and noninvasive biomarkers has already been demonstrated in several studies. Mitchell et al. published that prostate-specific antigen (PSA) is associated with exosomes in urine of patients with prostate cancer [47]. Other studies investigating EVs in relation to cancer showed that (I) vesicles from ascites of patients with colorectal cancer (CRC) may provide information on tumor development [48]; (II) claudin-containing exosomes in peripheral blood are associated with ovarian cancer [49]; (III) patients suffering from non-small cell lung cancer (NSCLC) can be distinguished from matched controls by a 30-marker model by phenotyping of EVs in plasma [50]; (IV) EpCAM-positive exosomes from NSCLC patients present increased levels of IGF-1R compared to healthy controls [51]. EVs have also been associated with neurological disorders like Alzheimer's disease. For instance, exosomes in cerebrospinal fluid of patients with Alzheimer's disease contain tau phosphorylated at Thr181, which is an established biomarker of this disease [52]. Furthermore, EVs have been investigated in relation to inflammatory and autoimmune diseases. Studies have shown that patients with systemic lupus erythematosus (SLE) and rheumatoid arthritis (RA) presented elevated levels of MVs and that these levels correlated with disease activity [53–55]. In addition, specific proteins, including galectin-3-binding protein (G3BP), and distinct groups of proteins, like immunoglobulin and complement components, were found to be enriched in these MVs [53, 54].

#### 1.2.2. Therapeutic Potential of EVs

In addition to the diagnostic potential of using EVs as biomarkers, EVs also hold a therapeutic potential, which has been exploited in the fields of vaccines and regenerative medicine. In relation to cancer, EVs have proven effective as potent inducers of antitumor immune responses. Clinical trials based on isolates of EVs have been used for treatment of different cancer types, including melanoma, NSCLC, and CRC [56–58]. For the melanoma and the NSCLC patients, autologous dendritic cells (DCs) were loaded with tumor-specific antigenic peptides and EVs from these cells were subsequently isolated and used for vaccination [56, 57]. Regarding the CRC patients, EVs were isolated from ascites and administered to patients in combination with treatment with granulocyte-macrophage colony-stimulating factor (GM-CSF) [58]. For regenerative purposes, EVs from mesenchymal stem cells (MSCs) have been tested in multiple disease models revealing that such EVs have the capability to neutralize myocardial ischemia and reperfusion injury [59], facilitate repair of kidney injury [60], and improve functional recovery in neurological diseases [61–64]. The regenerative potential of EVs exploits the fact that unmodified EVs can function as surrogates in place of the producing cell [65]. Importantly, compared to an analogous use of cells, exosomes are more stable and entail a reduced risk of immune rejections following* in vivo* allogeneic administration [66]. Furthermore, the inherent ability of EVs to carry cargo combined with the specific cellular targeting facilitates the use of EVs in therapeutic drug delivery. In this case, EVs are engineered for therapeutic delivery of nonnative cargo, for example, nucleic acids or medical drugs, and in some cases also engineered to display specific ligands that enable targeting of a particular tissue or cell type [65]. Indeed, targeting to specific recipient cells may reduce off-targets effects. Ohno et al. showed that EVs engineered to present a fusion protein of the transmembrane domain of platelet-derived growth factor receptor and the GE11 peptide could deliver a tumor suppressor miRNA (let-7a) to epidermal growth factor receptor- (EGFR-) expressing breast cancer cells [67]. Similarly, EVs from DCs were engineered to present a fusion protein of Lamp2b and the neuron-specific rabies virus glycoprotein peptide. Purified EVs were subsequently loaded with siRNA towards *β*-site amyloid precursor protein cleaving enzyme 1 (BACE1), which is a strong therapeutic target in anti-Alzheimer's treatment. Intravenous injection of these EVs in mice elicited high levels of mRNA and protein knockdown of BACE1 [68]. A study by Tian et al. demonstrated that DC-derived EVs engineered to present a fusion protein of Lamp2b and an *α*v integrin-specific peptide could deliver doxorubicin, a chemotherapeutic, to tumor tissue in mice, resulting in inhibition of tumor growth [69].

## 2. Characterization of EVs with Antibody-Based Assays

The following section focuses on a number of different antibody-based assays for profiling the protein composition of EVs. Common to all of these techniques are the use and dependence on antibodies. The list of antibody-based assays reviewed here is not absolute but covers both well-known platforms and recently developed technologies used for EV analysis.

### 2.1. Possibilities and Challenges with Antibody-Based Assays

A key step for development of robust antibody-based assays is the availability of highly specific antibodies that bind their target with high affinity. In fact, antibody affinity is the most important and limiting parameter for performing successful immunoassays [70]. Antibodies with high affinity provide both a better sensitivity and a larger dynamic range of the assays [71].

One advantage of several antibody-based assays is the ability to perform multiplexed phenotyping of EVs. Multiplexed protein profiling of EVs provides simultaneous information about multiple biomarkers, which increase the power of discrimination. A high power is likely essential in the search for new diagnostic and/or prognostic biomarkers. Thus, multiplexed antibody-based assays hold great potential for delivering data of diagnostic and prognostic value. However, multiplexed antibody-based assays may be constrained because of false positive signals generated by unspecific binding of antibodies (cross-reactivity) [72, 73]. Higher multiplexing levels are desirable for higher assay throughput in biomarker screening and yield a higher power, but the risk of unspecific binding increases exponentially with the level of multiplexing [72]. Antibodies validated for singleplex immunoassays may display cross-reactivity with other proteins in the multiplex platform, highlighting a need for application-specific validation of the antibodies [70, 74]. In addition, it is highly relevant to manage the assay sensitivity in order to provide acceptable dynamic ranges for each of the multiplexed proteins [70].

One limitation with antibody-based assays is the dependence on commercially available antibodies. The list of such antibodies is long in species like human and mouse but in cases where other species are of interest the access to commercial antibodies is at present more limited. In addition, antibodies may not even be available for novel candidate biomarkers. In spite of the above-mentioned limitations, antibody-based assays provide important information concerning the protein composition of the EVs investigated. In comparison to other technologies that merely provide information on size and numbers of EVs, for example, nanoparticle tracking analysis (NTA) and scanning ion occlusion sensing (SIOS), antibody-based assays provide information about features like the cell of origin and the cellular target, which can be translated into hypotheses regarding the functionality of the investigated EVs. Thus far, protein profiling of EVs has been restricted by the lack of widely accepted subtype-specific EV markers. Discovery of such markers would enable the inclusion of a common positive control and provide a direct identification of the subtype of EVs investigated. Moreover, such markers would also be helpful in differentiating the functions of the different EV subtypes.

### 2.2. Antibody-Based Assays for Phenotyping of EVs

#### 2.2.1. Flow Cytometry

**Figure 2:**
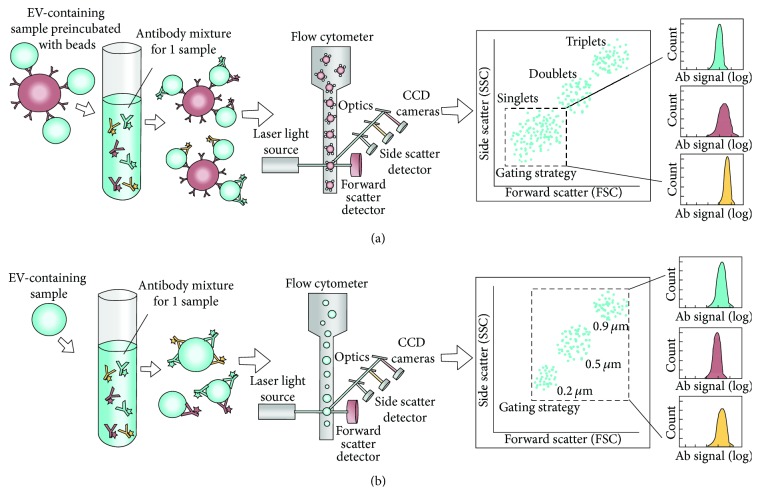
Schematic view of flow cytometric phenotyping of EVs. (a) Detection of bead-associated EVs. The EVs can be absorbed onto micron-sized beads, either directly or by antibodies coated to the beads. The latter approach causes a preselection of a subtype of EVs, based on the coating antibody. Subsequently, the absorbed EVs can be phenotyped by staining with selected, fluorophores-conjugated antibodies. The instrumental setup of lasers and optics allows for the detection of the EV-associated fluorescence. Moreover, the size of the beads facilitates detection with conventional flow cytometers (LOD ~ 300 nm). The gating strategy includes the creation of a singlet gate in a forward scatter (FSC) versus side scatter (SSC) plot, thus excluding aggregates. With this analysis, the relative amount of each investigated marker can be obtained for the entire population of bead-associated EVs. (b) Detection of individual EVs. The workflow for the flow cytometric phenotyping of individual/single EVs shares similarities to that described for bead-associated EVs. However, several differences also exist. First, there is a frequent requirement for a highly pure and enriched EV preparation prior to antibody labeling, especially when analyzing exosomes. Next, dedicated flow cytometers are used, lowering the LOD to approximately 150 nm. The use of SSC (in log scale) is often used as trigger to set the threshold for detectable events. However, labeling of all EVs with a fluorescent dye, with a subsequent use of this fluorescence signal as trigger, can serve as an alternative identifier. This may aid in increasing the signal-to-noise ratio, which is highly relevant for FCM analysis of EVs. The gating strategy involves setting a predefined EV gate based on the analysis of beads with a known size (0.2, 0.5, and 0.9 *μ*m). This both places the relevant gate and allows for a size evaluation of the EVs. The relative amount of each marker present on the EVs can subsequently be evaluated more precisely than for the bead-associated EVs, revealing the heterogeneity of EV phenotypes. Furthermore, enumeration of the EVs is possible by addition of a known amount of fluorescent beads or by analyzing an absolute volume of EV sample.

Currently, flow cytometry (FCM) is the most widely used technique for phenotyping of EVs in a clinical setting [75, 76]. It facilitates high throughput and multiparametric analysis of individual particles in a suspension, including cells and EVs. The particles are focused hydrodynamically and pass a laser beam, after which the scattered light and particle-associated fluorescence can be detected [77]. In this context, fluorochrome-conjugated antibodies can be used to target antigens on the EVs, thus enabling phenotyping of these vesicles (Figure 2) [78]. Moreover, a size distribution of the EVs is inherently obtained with FCM, while enumeration is also possible by addition of a known amount of fluorescent beads or by using an absolute volume analysis [79]. Hence, the method can be both qualitative and quantitative.

The EVs can be phenotyped either individually (Figure 2(b)) or after preabsorption to antibody-coated beads (Figure 2(a)). For both setups, EVs can be phenotyped by staining with selected, fluorophore-conjugated antibodies after which the samples are ready for analysis. The acquisition of EVs must be performed at low flow rates and at optimized concentrations to avoid coincident occurrence, where several particles are detected simultaneously [24, 75, 77]. Nevertheless, acquisition is relatively quick [79]. A major issue for FCM analysis of EVs is the low signal-to-noise ratios in the relevant detection area, relating both to the small size of the vesicles and to the relatively low fluorescence signals detected, due to low antigen density [75]. Therefore, it is crucial to reduce the background from nonvesicular contaminants, as well as performing stringent gating. A predefined EV gate is most commonly determined on the basis of a mixture of beads with different submicron sizes (Figure 2(b)). Although smaller sized beads (~100 nm) can be detected, the lower refractive index of EVs impairs the detection of EVs in this area [75].

FCM provides a limited ability for multiplex biomarker analysis. On a routine basis a range of six to 11 markers can be analyzed simultaneously, but the number of markers may be even higher depending on the particular flow cytometer used for the analysis [80]. In addition, FCM is said to be high throughput but each sample is stained and analyzed separately. Still, approximately 20 samples can be processed and analyzed within a few hours. A very common sample type for FCM is plasma [24, 75, 76, 81, 82], although EVs from several other media, including cell culture supernatant (CCS) [82–85], urine [86], and cerebrospinal fluid [29], have also been employed for phenotyping of EVs using FCM. For plasma, the use of 50–100 *μ*L of sample has been reported [24, 75], as well as a concentration range of 1 × 10^5^–1 × 10^6^ EVs/*μ*L [75]. Several protocols require extensive centrifugation before EV analysis [83, 84, 87], but examples of less comprehensive sample preparation can also be found [24, 29, 75, 76]. As previously mentioned, the isolated EVs can subsequently be phenotyped either individually or as bead-associated (Figure 2). The latter approach has often been necessary with the smallest EVs, since the estimated lower limit of detection (LOD) for conventional flow cytometers is close to 300 nm [78, 79]. However, with this approach the phenotypic heterogeneity of EVs cannot be fully appreciated. The next generation of flow cytometers, dedicated to submicron analysis, has changed the LOD to 150–190 nm [79]. However, using continuously optimized protocols combined with the newest technology, multiplexed FCM analysis of EVs, in terms of both marker and sample number, is highly relevant in the clinic [75, 79]. As mentioned, flow cytometry is the most widely used technique for assessment of EVs in clinical settings. One example is a prospective study on MVs from different groups of patients showing specific MV signatures associated with the different diseases (CRC, pancreatic cancer, inflammatory bowel, or pancreatic diseases), which may be used as a means of differentiating such cancer patients from associated inflammatory diseases [88].

#### 2.2.2. EV Array

The EV Array is based on the technology of protein microarray. It is capable of detecting and phenotyping EVs from unpurified starting material in a high throughput manner [89, 90]. The technology was developed to do multiplexed phenotyping of EVs in an open platform. Protein microarrays are well accepted as powerful tools to search for antigens or antibodies in various sample types [91, 92]. The advantage of protein microarray is that large numbers of proteins can be tracked in parallel; it is a rapid and highly sensitive method consuming only small quantities of samples and reagents.

**Figure 3:**
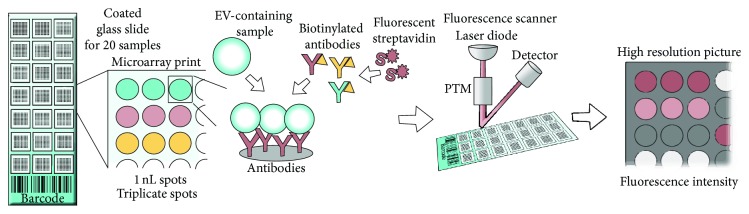
Schematic view of the EV Array. A basis for the procedure is epoxy-silane coated slides printed with 21 identical antibody microarray prints (for 20 samples and one control). Each antibody is printed in triplicate. EV-containing samples are applied and the EVs are captured onto the slides depending on the presence of surface antigens. Detection is performed by addition of biotin-labeled detection antibodies against the exosomal markers CD9, CD63, and CD81 followed by fluorescently labeled streptavidin. A fluorescence scanner is used to get a high resolution picture from which the fluorescent signals for each antibody spot can be quantified.

In the EV Array spots of capturing antibodies are printed on standard epoxysilane coated microarray slides in a customized spot setup using microarray printing technology (Figure 3). Following addition of EV-containing samples, the captured vesicles are detected using a cocktail of biotinylated antibodies against the tetraspanins CD9, CD63, and CD81 that are known as antigens present on exosomes in general [93–95]. Fluorescently labeled streptavidin is subsequently used to determine the amount of EVs captured on each individual microarray spot (Figure 3). To detect the fluorescence signals a microarray scanner or high resolution gel scanner with a microarray adaptor is needed.

The EV Array provides a relative quantification of the amount and the phenotype of EVs and can investigate up to 60 protein markers simultaneously. Currently, the method is optimized to be a high throughput analysis with 20 samples analyzed simultaneously on each microarray slide. The most common used sample type for the EV Array is plasma, but EVs from CCS, urine, ascites, and cerebrospinal fluid have also been employed for phenotyping of EVs. Fresh or frozen plasma samples are analyzed directly without preanalytical purification steps and only 10 *μ*L of plasma is needed. For each microarray spot (~1 nL), only 2.5  ×  10^4^ exosomes were required for a detectable signal [89]. The EV Array provides multiplexed phenotyping of EVs in 2 days.

The EVs captured on the EV Array are detected by a cocktail of antibodies against CD9, CD63, and CD81, indicating that the array is optimized for analyzing exosomes [89]. The technology of the EV Array leaves the possibilities open to change the detection antibodies in order to investigate other populations or subpopulations of EVs, for example, TF-bearing vesicles. One example in which the EV Array was transformed with a clinical perspective is published by Jakobsen et al. [50].

#### 2.2.3. Surface Plasmon Resonance Imaging (SPRi) Combined with Antibody Microarray

**Figure 4:**
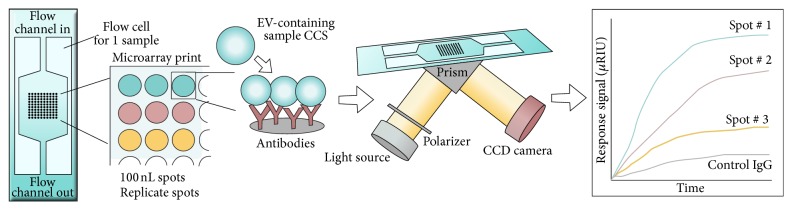
Schematic view of SPRi in combination with antibody microarray. Different antibodies are printed in replicate on a gold chip and EVs are introduced to the chip with a constant flow via a multichannel flow cell placed on top of the chip. EVs are captured based on the presence of surface antigens. The optical path from the laser passes through the coupling prism at a fixed angle of incidence and the reflection of light from the SPR-active gold surface is recorded by a charge-coupled device (CCD) camera. Capturing of EVs, by the antibodies on the chip, results in changes in the refractive index leading to changes in the reflection intensities monitored by the CCD camera. Changes in the reflection intensities are presented as resonance signal versus time.

The platform of surface plasmon resonance imaging (SPRi) in combination with the protein microarray technology was developed to facilitate sensitive, real-time, and label-free relative quantification of EVs [96]. The combined setup of SPRi and the antibody microarray platform enables investigation of EVs in relation to both size (SPR is mass sensitive) and phenotype (antibody detection). In the combined setup, different antibodies are printed on a gold chip and EVs are introduced to the chip with a constant flow via a multichannel flow cell placed on top of the chip (Figure 4). Consequently, EVs are captured based on the presence of vesicular surface antigens. Upon illumination of the chip, light passes through the coupling prism at a fixed angle of incidence and the changes in the reflection of light from the SPR-active gold surface are transformed into the refractive index changes resulting from EV binding. Both reflection detection and surface imaging are recorded by a charge-coupled device (CCD) camera and the average reflectivity changes of selected areas are plotted as a function of time (Figure 4) [97].

As with the EV Array, the SPRi assay can track several proteins in parallel. The platform is rapid and highly sensitive and consumes small quantities of samples and reagents. For high throughput multiplex measurements, a multichannel fluidic cell system is required, along with the detection system integrated with optics consisting of a laser diode and a CCD camera. The current SPRi, coupled with the microarray technique, is optimized for EVs (mean diameter of approximately 70 nm) from CCS but could very likely be extended to EVs from bodily fluids. The sample is injected at a rate of 5 *μ*L/s, so if the sample is applied for 400 s, approximately 2 mL of sample is used for each analysis [96]. Cell culture supernatants were subjected to a 3-step centrifugation prior to analysis, but no further preanalytical purification was needed. Titration experiments for the limit of detection were not reported, but a study by Zhu et al. described detection of EVs by anti-CD9 and anti-CD41b in CCS from 1 × 10^8^ cells [96]. The SPRi assay enables multiplexed phenotyping of EVs in <30 min.

#### 2.2.4. Nanoplasmonic Exosome Assay (nPLEX)

The nPLEX is also a SPR-based assay for label-free, high throughput EV protein analysis. It is developed for relative quantification of the amount and the phenotype of EVs [98, 99]. It is based on optical transmission through periodic nanoholes rather than total internal reflection as used in conventional SPR systems. As the mass sensitivity of the SPR technique is combined with the inclusion of antibodies, EVs are defined by both size and phenotype.

**Figure 5:**
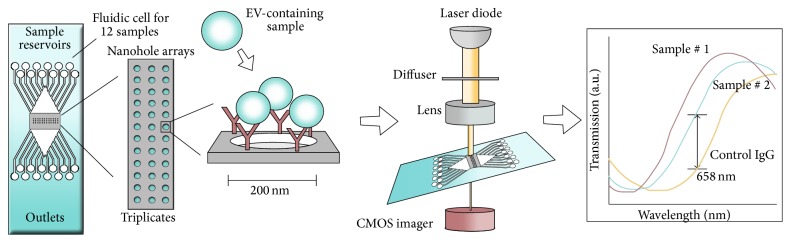
Schematic view of the nPLEX platform. Antibodies are immobilized on the nPLEX chip (chip with periodic nanoholes patterned in a gold film) and EVs are introduced to the chip via a 12-channel fluidic cell placed on top of the nPLEX chip. EVs are captured based on the presence of surface antigens. The optical path from the laser passes through the nPLEX chip which is located on top of the imager and the light intensities transmitted through the nanoholes arrays are recorded in parallel by the complementary metal oxide semiconductor (CMOS) imager. Light illumination to the nanohole array excites strong electromagnetic fields, which lead to optical transmission. EV binding to the nanohole surface (via antibodies) can redshift the optical transmission peaks, which can be monitored by measuring intensity changes at fixed wavelength (658 nm).

In the nPLEX system periodic nanoholes are located in specific patterns in a gold film. Initially, antibodies are immobilized in the nanoholes on the nPLEX chip, and EVs are captured based on the presence of the selected EV surface antigens (Figure 5). Light illumination through the nanohole arrays excites strong electromagnetic fields, called surface plasmons on the surface, which lead to surface plasmon-mediated extraordinary optical transmission. The transmission spectral peak positions are highly sensitive to the refractive index on the nanohole surface, and EV binding to the nanohole surface (via antibodies) shifts the optical transmission peaks. These shifts can be monitored by measuring either wavelength shifts in light spectrum or intensity changes at fixed wavelengths. A portable imaging system has been developed to be implemented in the clinic. The portable imaging system is combined with the nPLEX chip consisting of a laser diode and a complementary metal oxide semiconductor (CMOS) imager (Figure 5). The nPLEX sensor is located on top of the imager and the light intensities transmitted through the nanoholes are recorded in parallel by the imager [100, 101].

For high throughput analyses a 12-channel fluidic cell is placed on top of the nPLEX chip enabling phenotyping of either 12 markers in one sample or one marker in 12 samples. So far the nPLEX has analyzed EVs from ascites samples and CCS but could readily be extended to EVs in other bodily fluids. In addition, the current method is optimized for investigation of exosomes. Im et al. established a quantitative assay protocol that reports both EV concentrations and EV protein levels (average level of target protein per EV), while consuming approximately 1 *μ*L of sample per channel [98]. Prior to EV analysis, ascites were filtered through a 0.2 *μ*m membrane filter. In addition, CCS need further purification (differential centrifugation) prior to analysis. Titration experiments determined the nPLEX LOD to be approximately 3 × 10^3^ EVs. The observed sensitivity was 10^4^-fold higher than that of western blot analysis [99] and 10^2^-fold higher than that of chemiluminescence ELISA [98]. The entire nPLEX array provides multiplexed phenotyping of EVs in <30 min.

#### 2.2.5. Micro-NMR

Micro-NMR (*μ*NMR) is a highly sensitive and rapid analytical technique developed for phenotyping of circulating EVs from blood samples [99]. Briefly, EVs, introduced onto a dedicated microfluidic chip, are labeled with target-specific magnetic nanoparticles (MNPs) and detected by a miniaturized micronuclear magnetic resonance (*μ*NMR) system [102, 103]. Based on both size (vesicles 50–150 nm) and immunoaffinity (CD63) the method provides a relative quantification of the amount and the phenotype of EVs.

**Figure 6:**
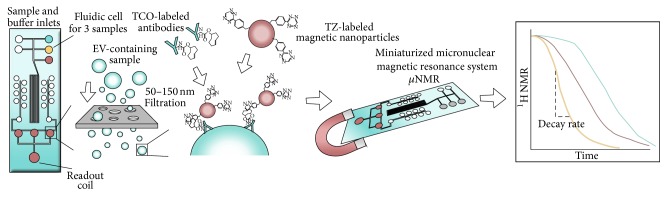
Schematic view of the micro-NMR technology. Three samples can be applied onto the prototype of the microfluidic cell together with buffer and antibodies. First step in the procedure is a filtration through pores of 50–150 nm after which EV surface antigens are targeted with antibodies labeled with trans-cyclooctene (TCO). For detection 1,2,4,5-tetrazine (TZ) labeled magnetic nanoparticles (MNP) are added and captured EVs are detected with the miniaturized *μ*NMR system. The *R*_2_ relaxation is equivalent to the amount of MNPs and thereby the amount of surface antigen present in the sample.

For detection of EVs by microfluidic *μ*NMR, the EVs are labeled with target-specific MNPs by immune-targeting of specific markers, for example, CD63 (Figure 6). The magnetic labeling makes the EVs superparamagnetic, which results in faster decay of the ^1^H NMR signal. The decay rate (*R*_2_) is proportional to the MNP concentration, thus enabling quantification of the targeted vesicular surface protein. The technique uses a two-step bioorthogonal approach for MNP labeling that maximizes MNP binding. EVs are first targeted with antibodies modified with* trans*-cyclooctene (TCO) and then coupled with MNPs derivatized with 1,2,4,5-tetrazine (TZ) (Figure 6) [104]. The *R*_2_ relaxation was measured using Carr-Purcell-Meiboom-Gill pulse sequences. For the analysis, a prototype device was developed for a clinical setting. The device contains three essential components: (I) a chaotic mixer for mixing EVs with antibodies and MNPs, (II) a membrane filter for washing and concentrating, and (III) a microcoil for NMR detection. Together with the prototype device, a miniaturized NMR relaxometer is needed to perform the analysis [103].

So far the *μ*NMR platform does not offer multiplexing in relation to markers, but three samples can be applied to the fluidic cell simultaneously. Shao et al. described that preanalytical purification of plasma samples is required (filtration, 0.8 *μ*m filter, and centrifugation). Samples can be frozen prior to the EV isolation. The EVs analyzed were shown to have a typical size distribution ranging from 50 to 150 nm due to the cut-off sizes of membrane filtration. Following isolation (centrifugation), one *μ*L of the pelleted EVs is loaded onto the *μ*NMR device for analysis and the relative content of a single EV marker is determined within seconds. Signals were detectable down to ~10^4^ EVs [99]. The hand-sized relaxometer used for the analysis makes the method a useful diagnostic tool in future clinical settings.

#### 2.2.6. Bead-Based Microfluidic Assays

He et al. developed a microfluidic EV analysis platform that utilizes bead-based enrichment for phenotyping of EVs present in blood samples [51]. The platform is an integrated microfluidic approach that enables on-chip immunoisolation and* in situ* protein analysis of EVs. The assay provides a quantitative detection of surface and intravesicular proteins.

**Figure 7:**
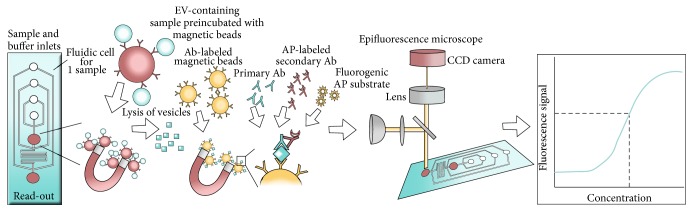
Schematic view of the bead-based fluidic assay. For this microfluidic assay, a prototype polydimethylsiloxane (PDMS) chip was developed. The device is designed with four inlets and a cascading microchannel circuit for sequential isolation and phenotyping of EVs. Plasma is premixed with antibody-labeled magnetic beads and introduced onto a PDMS chip, where bead-labeled EVs are magnetically retained. The EVs are subsequently lysed and the released proteins (surface and intravesicular antigens) are captured by antibody-labeled magnetic beads and detected by a sequential introduction of the primary detection antibody, the secondary antibody (alkaline phosphatase labeled), and a fluorogenic substrate (DiFMUP) for a sandwich immunodetection of the antigens of interest. An epifluorescence microscope equipped with a mechanical shutter and a CCD camera was used for chemifluorescence detection. A filter set (excitation 325–375, emission 435–485) was employed for detection. By the use of protein standards, the assay provides a quantitative detection of the protein of interest.

Plasma is premixed with antibody-labeled magnetic beads and introduced onto a prototype chip containing a cascading microchannel network allowing detainment of bead-labeled EVs (Figure 7). The EVs are subsequently lysed by incubation of the captured EVs in lysis buffer. The lysate then flows into a serpentine channel and antibody-labeled magnetic beads are injected from two side-channels to capture both surface and intracellular antigens. Captured antigens are subsequently magnetically retained and detected by a sequential introduction of primary antibodies, secondary antibodies (alkaline phosphatase labeled), and a fluorogenic substrate (DiFMUP) for a sandwich immunodetection of the antigens of interest. As antibody-labeled magnetic beads are used for both isolation and profiling of EVs, magnets are required for retaining bead-bound EVs in the microchannel network. In addition, a 4-syringe programmable pump system was used for controlled reagent delivery. For signal detection, an upright epifluorescence microscope equipped with a mechanical shutter and a CCD camera was applied in the described setup.

By the use of protein standards, the assay provides a quantitative detection of the protein of interest. As a proof-of-concept, the level of insulin-like growth factor 1 receptor (IGF-1R) and phosphorylated IGF-1R was determined in plasma samples from NSCLC patients. The study achieved quantitative detection of both proteins with a 100-fold greater sensitivity than the one achieved by commercial ELISA kits [51]. So far, this platform does not offer multiplexing, neither in relation to markers nor in relation to number of samples. He et al. only describe the use of plasma (30–150 *μ*L), but the method could probably be extended to EVs from other fluids. The immunocaptured EVs were shown to have a size distribution ranging from 40 to 150 nm, but other subtypes of EVs can most likely be captured if antibodies specific for these subtypes are used. A great advantage of this microfluidic platform is that it does not require preanalytical purification of EVs. In addition, the method provides a quantitative detection of surface, as well as intracellular antigens within 120 minutes.

## 3. Technical Summary

The above reviewed assays are all antibody-based but in spite of this similarity the assays are technically very diverse. Firstly, the requirements for preanalytical purification are different. So far it has been widely recognized that EV-containing preparations like CCS and plasma need purification prior to analysis. Unfortunately, isolation/purification of EVs has proven quite difficult. EV purification may be achieved by a variety of methods, including ultracentrifugation, size exclusion (e.g., filtration), immunoaffinity isolation, and microfluidic techniques. Many of the current isolation steps are time-consuming (e.g., ultracentrifugation) and may not even result in a pure preparation of a specific EV subtype [105, 106]. In addition, isolation may include a risk of damaging the EVs (e.g., filtration, polyethylene glycol (PEG) precipitation) [107] and may result in artifacts or loss of material and thereby loss of valuable information. Of the assays reviewed here, the EV Array, SPRi combined with the antibody microarray, and the bead-based microfluidic assay are optimized for analysis of EVs without a preanalytical purification step. With many unknown factors, still related to the different purification methods, it may be expected that future technical setups favor assays that do not require EV purification from the EV-containing samples. Secondly, the assays are optimized for different subpopulations of EVs. Flow cytometry currently works best with EVs larger than 300 nm, but as the technology evolves the possibility to investigate smaller size EVs arise. The other assays are mostly described in relation to exosomes, but several of them may, upon a few changes, provide the opportunity to investigate other subpopulations as well. Thirdly, the six assays differ in the ability to perform multiplex EV analysis. Multiplexing may refer to either the number of markers or the number of samples investigated in parallel. Assays like *μ*NMR and the bead-based microfluidic assay provide singleplex EV analysis in relation to markers. In relation to the number of samples investigated, the bead-based microfluidic assay only investigates one sample at a time, while *μ*NMR provides the opportunity to investigate 3 samples simultaneously. Flow cytometry and the nPLEX possess the ability to investigate a limited number of markers on one sample. However, the nPLEX setup may be reversed providing analysis of one marker in 12 samples. In addition, even though samples are processed and analyzed separately, the nPLEX platform is fast, thus providing the opportunity to process several samples in few hours. Likewise, flow cytometry offers the opportunity to process and analyze approximately 20 samples within a few hours. While the EV Array is performed with up to 60 markers, the SPRi assay does not state a limit regarding the number of markers. In relation to the number of samples, the EV Array possesses the ability to investigate 20 samples simultaneously, while the SPRi assay investigates one sample at a time. Regarding the output, the majority of the assays provide a relative quantification of the amount of EVs, as well as the level of the marker(s) of interest. The bead-based microfluidic assay stands out as being able to detect both surface and intravesicular proteins. In addition, the assay facilitates quantitative measurements of the markers of interest by including protein standards. Similarly, flow cytometry stands out as being the only techniques that facilitate enumeration of EVs (providing exact number of EVs) and a size distribution of the EV population.

When comparing the six techniques it is evident that some parts of the technological platforms are identical, but none of them are identical in overall structure. The characteristics of the assays are outlined in Table 1. Due to the technical differences within the six assays, it must be expected that the outcome differs and a particular analysis cannot directly be transferred from one technical platform to another. This limitation is one of the main factors that complicate the translation of basic EV research into a general clinical setting.

## 4. Future Technical Perspectives

The technologies described above exploit the specificity and sensitivity associated with antibody-based assays to identify biological and pathological fingerprints of EVs at the proteome level. Every one of these technologies has proved efficient for phenotyping of EVs and useful in the discovery of new biomarkers. However, new technologies constantly emerge providing improved setups in relation to features like throughput, number of markers investigated, and the amount of sample needed for analysis. A recently published study by Assarsson et al. describes a proximity extension assay (PEA) that alleviates some of the shortcomings of antibody-based assays [71, 72]. The assay is based on pairs of antibodies that are linked to oligonucleotides having slight affinity to one another. Upon antibody binding, the oligonucleotides are brought in proximity and can thereby be extended by a DNA polymerase, thus forming a new sequence that acts as a unique surrogate marker for the specific protein. Due to the proximity requirement for template formation, the inherent risk of antibody cross-reactivity is reduced [72, 73]. The assay is able to simultaneously measure 92 markers in 96 samples. So far, this PEA technique has not been assayed specifically on EVs but reports only on molecular biomarkers in plasma samples. However, the technique appears very promising as a future EV analysis platform. Similarly, Pla-Roca et al. describe an antibody-based microarray with a different approach for multiplexing called antibody colocalization microarray (ACM) [108]. In ACMs, both capturing and detection antibodies are physically colocalized by spotting to the same two-dimensional coordinate. In this way, mixing of detection antibodies is avoided, which reduces the risk of cross-reactivity. The technique has so far been optimized for 50 antibodies. Again, the ACM has not been assayed specifically on EVs but seems encouraging as a future assay for EV analysis.

The techniques presented in this review are clearly useful tools in the search for disease-specific biomarkers, but to fully harness the diagnostic and prognostic potential of EVs, several aspects remain to be delineated. The first is standardization; standardization relates to multiple areas of EV analysis including nomenclature, preanalytical conditions, and isolation procedures. Implementation of standardized protocols will facilitate unbiased comparisons of results from different laboratories [107]. Secondly, if EV analysis is to be employed in a diagnostic manner, it is important to select methods that ask for acceptable requirements in relation to the patient samples, apparatus, and, last but not least, methods that are cost-effective. Overall, future technologies should preferably facilitate EV analysis without the need for prepurification of patient samples, possess the ability to determine a large number of biomarkers simultaneously on a minimum of patient material, and provide high sensitivity as well as high throughput of samples. Altogether, the rapidly growing knowledge of the biology of EVs combined with the many technological advances set high expectations for future clinical applications of EVs.

## Figures and Tables

**Table 1:** Characteristics of the antibody-based assays reviewed here.

Technique	Purification	Optimized to analyse	Sample	Timeframe^*∗*^	Multiplexing (markers/samples)	Output
Flow cytometry	±centrifugation^**∗∗**^	EVs ≥ 300 nm	50–100 *µ*L	Hours	Limited/1	Size distribution, phenotype, enumeration

EV Array	None	Exosomes	≥10 *µ*L	Days	60/20	Relative quantification of amount and phenotype

SPRi microarray	None	Exosomes	5 *µ*L/s	Minutes	Several (Limit not indicated)/1	Relative quantification of amount and phenotype

nPLEX	Filtration ± centrifugation	Exosomes	12 *µ*L	Minutes	Prototype 1/12 or 12/1 (scalable)	Relative quantification of amount and phenotype

*µ*NMR	Filtration + centrifugation	Exosomes	1 *µ*L (pelleted EVs)	Minutes	1/3	Relative quantification of amount and phenotype

Bead-based microfluidic	None	Exosomes	≥30 *µ*L	Hours	1/1	Quantification of phenotype (surface and IC)

^**∗**^Preanalytical purification procedures not included.  ^**∗∗**^Ultracentrifugation and density gradient centrifugation are often required when exosomes are analyzed by flow cytometry; however, when analyzing MVs, preanalytical purification is not always performed.
